# 

**Published:** 1918-11-02

**Authors:** 


### German and Austrian Scientists Banned.

THE German and Austrian professors and scientists have all been removed from association with the Institute, and their names erased from the Register, by a unanimous vote at a general meeting of the Institute of Hygiene.

### Alexandra Day Awards.

ALEXANDRA DAY raised £23,875 this year, which is approximately £2,000 more than in 1917. At the request of the committee Queen Alexandra nominated a list of about a hundred to have divided amongst them £9,260, and the allocation of the balance of the fund was the work of the Distribution Committee. Some of the larger grants are as follows: The National Refuges for Homeless and Destitute Children, £1,000; the British Home for Incurables, Streatham, and the Church Army, £500 each; the London Hospital, Queen Victoria's Jubilee Institute for Nurses, and the Royal Surgical Aid Society, £300 each; the Salvation Army, the Alton Home and Hospital for Cripples, the Royal United Kingdom Beneficent Association, and St. Dunstan's Hostel, £250 each. It is stated that the remainder of the money is shared out with regard to local needs in the Metropolitan boroughs where collections were made. Queen Alexandra has thanked the Lord Mayor and the committee for the devotion shown by them in the administration of the Fund, and congratulated them upon the very marked success achieved. It will be noted that in the larger grants established voluntary hospitals do not come out well, yet we suspect a large proportion of the givers are under the impression that the appeal is primarily for these institutions.

### Passing Events and Topics.

### Agricultural Activity at Putney.

OVER ten tons of potatoes have been raised this year in the gardens of the Royal Hospital and Home for Incurables, Putney Heath. The hay crop was sold for £90.

### Peterborough War Memorial.

As a memorial of the war, a new hospital is to be erected at Peterborough, which is estimated to cost between £50,000 and £75,000.

### Matrons' and Sisters' Appointments.

ELLEN BADGER COTTAGE HOSPITAL, SHIPSTON-ON-STOUR.—Miss Elizabeth Gillies has been appointed nurse-matron. She was trained at the Glasgow Royal Infirmary, and has been matron of Bromyard Cottage Hospital.

INFECTIOUS DISEASES HOSPITAL AND TUBERCULOSIS HOSPITAL, NEAR SUNDERLAND.—Miss Ida C. Thompson has been appointed matron. She was trained at Bedford County Hospital and at the Royal National Hospital for Consumption, Newcastle, Co. Wicklow, and has been night superintendent nurse and second assistant matron at Queen Mary's Hospital, Carshalton.

ISOLATION HOSPITAL, SMITHY BRIDGE.—Miss M. L. Mensforth has been appointed sister. She was trained at the Keighley Union Infirmary.

MILITARY HOSPITAL, BETHNAL GREEN.—Miss M. E. Healey has been appointed ward sister. She was trained at Charing Cross Hospital, and has since been undertaking sister's duties.

ST. JOHN AUXILIARY MILITARY "A" HOSPITAL, MORECAMBE.—Miss S. Grace Nobbs has been appointed matron. She was trained at the General Hospital, Auckland, New Zealand, and has been matron of the General Hospital, Opotika, New Zealand; the General Hospital, Cook's Islands; Ramsgate Auxiliary Military Hospital, and of the Chigwell Auxiliary Military Hospital.

**NOTE.—Particulars of Appointments for insertion in this column will be welcomed.**

### Recent Official Publications.

(To be obtained from H.M. Stationery Office, Imperial House, Kingsway, W.C. 2. All prices quoted are postage free.)

Local Government Board for Scotland. Supplementary List of Charities from February 1 to December 31, 1917. 1½d.

Statutory Rules and Orders, 1918, No. 1,136. Public Health, England. Prevention of Epidemic, Endemic, or Infectious Diseases. Regulations (No. 2), September 6, 1918. 1½d.

National Health Insurance. Medical Benefit Regulations, 1918. Draft Regulations, September 20, 1918. 1½d.

Manual of Military Cooking and Dietary, 1918. 8d.

Local Government Board. Pauperism (England and Wales) Quarterly Statement, April to June 1918. 4d.

Local Government Board. Maternity and Child Welfare Grant, Hospitals and Homes. 1½d.

Reports of the Local Government Board on Public Health and Medical Subjects. Further Reports and Papers on Epidemic Poliomyelitis. 1s. 2d.

National Health Insurance. Special Report Series No. 20. A Study of Social and Economic Factors in the Causation of Rickets. Medical Research Committee. 2s. 3d.

Ministry of Reconstruction. Advisory Council. Women's Housing Sub-Committee. First Interim Report. 1½d.

Lunacy and Mental Deficiency. Fourth Annual Report of the Board of Control for the Year 1917. Part 2. 4s. 2d.

### Forthcoming Events.

ARMY AND NAVY MALE NURSES' CO-OPERATION.—Eleventh annual general meeting, at 52 Welbeck Street, Monday, November 4, at 4 P.M.

NATIONAL HOSPITAL FOR THE PARALYSED AND EPILEPTIC.—Owing to the prevalence of influenza, the commemoration of Founders' Day, fixed for November 4, has been postponed.

### The College of Nursing
### (Limited by Guarantee).

LIVERPOOL CENTRE: SYLLABUS OF LECTURES.

THE following lectures will be given in the Lecture Theatre of the Liverpool Royal Infirmary, commencing at 6.15 P.M.: NOVEMBER 14, "Nursing of Diseases of the Eye," T. H. Bickerton, Esq., L.R.C.P., M.R.C.S.; DECEMBER, "Venereal Disease," Mrs. Powell Bigland, M.D., D.P.H.; JANUARY 1919, "Wounds and their Treatment," W. Thelwall-Thomas, Esq., Ch.M., F.R.C.S.; FEBRUARY, "A Nurse as an Officer of a Public Authority: Her Duties and Opportunities," W. Allen Daley, Esq., M.D., D.P.H., M.O.H., Bootle; MARCH, "X-ray and Orthopædics," Major Thurstan Holland, L.R.C.P., M.R.C.S.; APRIL, "The Future of the Nurse after Training," Miss Gertrude Cowlin, Organising Secretary, College of Nursing. Members of the College will be admitted to the lectures free. Tickets for non-members may be obtained from Miss Golding, price 3s. the series.

### A Novel Money-raising Scheme.

THE Mayor of Banbury started a trail of pennies from Banbury Town Hall, and by four o'clock the same afternoon two miles of kerbstones, guarded by wounded soldiers, had been traced with coins. About £100 was raised by this novel effort in aid of the Red Cross Funds.

